# Retraction: Comparison of High-Statin Therapy vs Moderate-Statin Therapy in Achieving Positive Low-Density Lipoprotein Change in Patients After Acute Coronary Syndrome: A Randomized-Control Trial

**DOI:** 10.7759/cureus.r51

**Published:** 2022-03-17

**Authors:** Chamithra D Rupasinghe, Theodosios Kantas, Rohail Sani, Natalia M Avendaño Capriles, Ramil dadabhoy, Afreenish Gul, Camilo Andrés Avendaño Capriles, Noman Khurshid Ahmed, Sohaib Tousif

**Affiliations:** 1 Internal Medicine, Manipal College of Medical Sciences, Pokhara, NPL; 2 Department of Surgery, General Athens Hospital, Athens, GRC; 3 Medicine, Shifa College of Medicine, Islamabad, PAK; 4 Medicine, Universidad del Norte, Barranquilla, COL; 5 Medical College, Ziauddin University, Karachi, PAK; 6 Internal Medicine, Ziauddin University, Karachi, PAK; 7 Foundations of Clinical Research (FCR) Program, Harvard Medical School, Boston, USA; 8 Internal Medicine, Aga Khan University Hospital, Karachi, PAK; 9 Medicine, Ziauddin University, Karachi, PAK

This article has been retracted due to the unknown origin of the data, lack of verified IRB approval, and purchased authorships. While not listed as an author, it was discovered that Rahil Barkat wrote and coordinated the submission of this article. Mr. Barkat was involved in data theft and misuse in two recently published Cureus articles, which have since been retracted.

As the origin of this article’s data and verified IRB approval cannot be confirmed, we have made the decision to retract this article. Cureus has confirmed that the co-authors were asked by Mr. Barkat to proofread the article and provide payment in exchange for authorship. (Proofreading is an insufficient contribution to warrant authorship as defined by ICMJE.) These payments were made in the guise of “editing fees” but greatly exceed any editing fees paid to Cureus. While these authors may have been defrauded by Mr. Barkat, they remain complicit due to their lack of honest contributions to the article.

